# Well-Designed Food Governance as Psychological Mechanism of Consumer Perceptions in the Context of Tourism Poverty Alleviation

**DOI:** 10.3389/fpsyg.2020.590816

**Published:** 2021-02-11

**Authors:** Guo-qing Huang, Kuen-Lin Lin

**Affiliations:** ^1^College of Economic and Management, Southwest University, Chongqing, China; ^2^Cheng Shiu University, Kaohsiung, Taiwan

**Keywords:** food governance, tourism poverty alleviation, psychological remedy, developing countries, micro- foundation

## Abstract

Poverty is a challenge leading to food insecurity in people's minds. This article discusses food governance as a psychological mechanism to facilitate the sense of wellness in people's minds in the context of tourism poverty alleviation. Mainly, we argue that, when a government is implementing tourism poverty alleviation, not only are economic efforts, but also positive psychological feelings are required. We, thus, argue that sound food governance may increase the sense of wellness in the minds of people as food consumers by increasing food safety and security. This perspective paper contributes by explicating the influences of macrolevel governance design of safer and more secure food systems on people's psychological wellness, especially against the background of tourism poverty alleviation in developing countries.

## Introduction

Poverty is a critical challenge that leads to food unsafety and insecurity in both developing and the least developed nations. Globally, food insecurity/unsafety as a direct product of poverty leads to malnutrition and low quality of life. Accordingly, existing food security interventions targeting tourism poverty alleviation are discussed in the context of practical actions and positive measures. In this regard, research studies consistently point out environmental management, increased farm yields, youth engagement in agricultural production, and investment in job creation. Based on this, biological, chemical, and economic aspects of food security/safety for tourism poverty alleviation have dominated governance discourses.

However, psychological concerns cannot be ignored beyond those important perspectives above. In addition to the resulting problems mentioned above, poverty may also challenge the government and people by impeding significant cognitive and psychological development (e.g., less chance for intelligence training or low self-esteem) (OOO). Put differently, when designing and implementing governance mechanisms for poverty intervention via food security/safety improvements, psychological mechanisms and logics should also be discussed systematically.

Consumer psychology and behaviors should also be incorporated as a central issue to solve problems of poverty and food insecurity through enhanced food governance [for details, see Wu et al. (2018), Chen and Wu (2019)]. Despite their existence, less is said about how a better “food” governance at enterprise/industry/national levels is a remedy for tourism poverty alleviation through psychological mechanisms that alter consumer perceptions and behaviors. For this reason, this is a conceptual paper that addresses the relationships of the chain of food governance at enterprise/industry/nation levels through psychological interventions that remedy poverty levels in developing economies.

**Figure 1:**
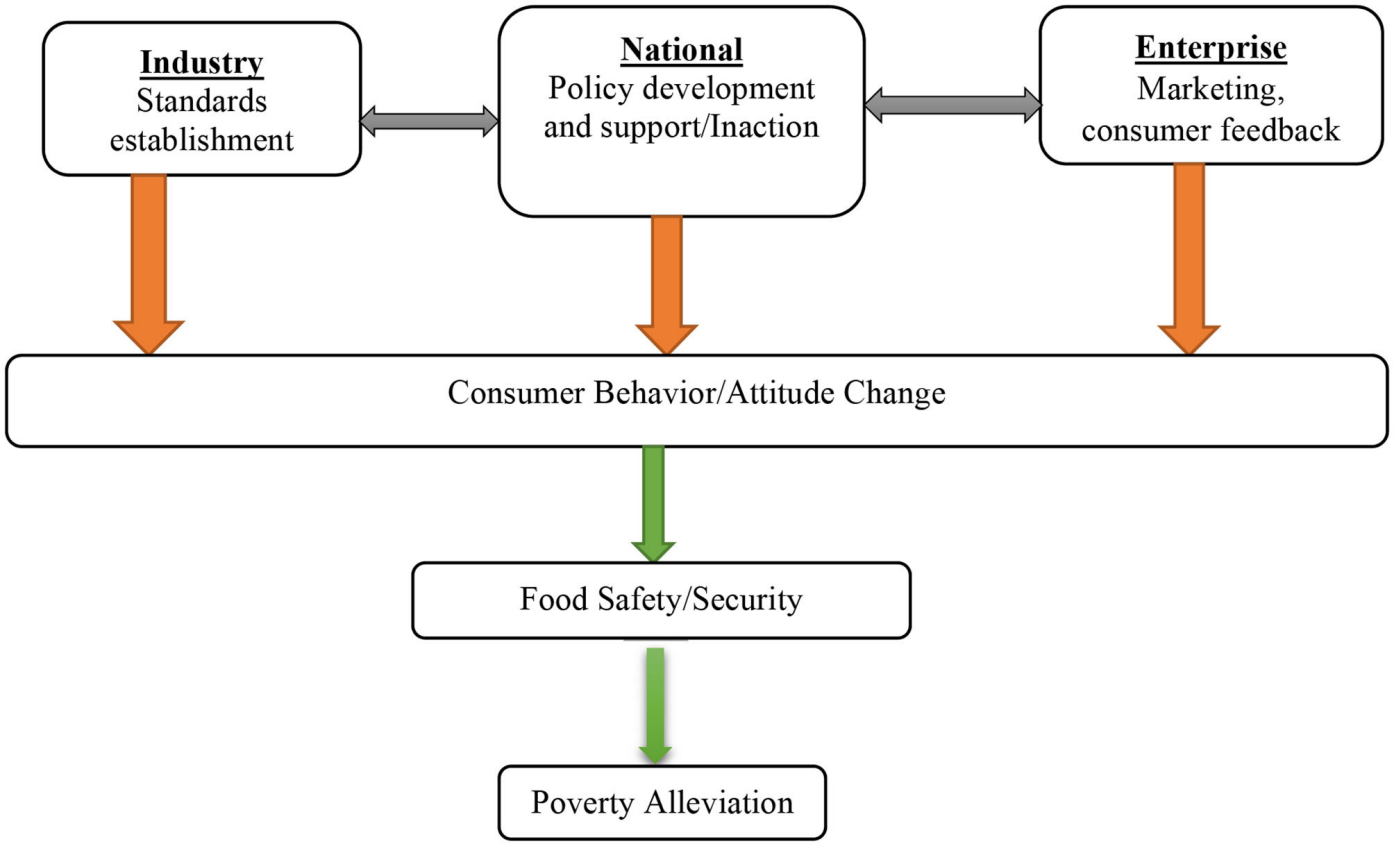
Conceptual framework (the orange lines mean “psychological mechanisms' influences” and the green lines mean “facilitation”).

Against this background, every interventional level is tackled independently while also looking at existing linkages that explain the psychological mechanisms that can alter consumer perceptions/behaviors for improved food governance. In Figure 1, a comprehensive schema is sketched to lead our following discussions. The study's aim and angle is 2-fold: (a) to delineate and investigate the interplay between industry, enterprise, and the national food governance framework and (b) to delineate and investigate the impact of the interplay between industry, enterprise, and the national food governance framework on consumers' perception and, thus, consumer behavior.

## Literature Review

### Correlation Between Poverty and Food Security

Poverty is directly connected to food insecurity, especially in a down time such as the COVID-19 pandemic period. Källestål et al. (2020) analyze poverty-related problems in four municipalities in Northern Nicaragua using the Unsatisfied Basic Needs (UBN) index. The study explores multiple dimensions of poverty based on the capability approach using data mined from the Cuarto Santos Health and Demographic Surveillance database using the K-means algorithm. The capability approach gives beneficiaries the right to determine an alternative life they would prefer subject to interventions. Accordingly, the study finds that fairly rich households, based on the UBN index, had modern lifestyles based on subjective choice, but poor households were food insecure. In this regard, this study establishes a direct connection between poverty and food insecurity.

In sub-Saharan Africa, food security and agricultural productivity among small-scale farmers determine food security and the livelihoods of rural households. With reference to the aforementioned, Mutea et al. (2019) find that solving food security challenges in rural agro-dependent communities requires an appreciation of the link between food insecurity and livelihood. In particular, the study relies on the food security index to determine food security and livelihood drivers. The aforementioned include variables, such as food consumption score, household dietary diversity score, coping strategies index, household food security access index, and months of adequate household provisioning. Upon analysis, they find that 32% of households under the survey were food secure, and the rest were insecure. Food security was dependent on household productive tool ownership, off-farm incomes, own-food consumption, and agro-ecological factors as well as pest destruction. Notably, semi-humid and semi-arid conditions contribute to food security with all factors constant although humid and semi-arid ecological climatic circumstances negate food security.

To stress the point further, studies demonstrate that agro-ecological conditions impact food security and malnutrition in children under the age of 5 years from low-income backgrounds. Chakona and Shackleton (2018) investigate the connection between malnutrition risks in children under 5 years old from poverty-stricken households as a result of problems related to food insecurity. Using the Household Dietary Diversity Score (HDDS) and Household Food Insecurity Access Scale (HIFAS), the study finds that dietary diversity increases with agro-ecological potential. On the other hand, HIFAS measurements increase with a low agro-ecological gradient. Based on this study, enhanced food production enhances food security through dietary diversity, which then reduces malnutrition risk among children under the age of 5 years old.

Cognitive maladjustments and attitude toward poverty depend on childhood food security experiences. In this regard, Hermida et al. establish that low socioeconomic status directly impacts rural poor children compared to their urban counterparts. As such, the study finds that children in poverty perform poorly in “executive functions and non-verbal communication intelligence.” As such, they posit that policy interventions should target childhood cognitive development among children from poor backgrounds. Moreover, Lipina and Evers (2017) argue that cognitive development in children is dependent on the biological, psychosocial, and socio-cultural factors related to poverty. Consequently, they suggest that neuroscience can play a critical role in understanding cognitive development in children and their susceptibility to poverty.

### Food Security and Tourism Poverty Alleviation Interventions

Government policy geared toward vulnerable portions of the population is an immediate intervention to stave off food insecurity across the world, and the quality thereof, as well as health, is equally integral. Accordingly, Philip et al. sought to investigate the long-term implications of food aid on health and well-being with a specific focus on the quality of affluent Israel food pantries. Based on the Healthy Portions Score, the study sampled 105 beneficiaries of food aid from 16 food baskets across the country. The study finds that social intervention through food aid in terms of quality of the recipient diet is critical in policy making. Notably, interest in this regard is on the consideration of diet and quality in the food security policy.

Low socioeconomic status increases food consumption compared with food secure individuals when provided with free provisions. Accordingly, Godsell et al. induced low socioeconomic status among 123 adult participants and a control group. They then subjected them to the availability of free snacks, and food-secure participants consumed less compared with the food-insecure participants. As a result, the sense of deprivation in poor food consumption leads to unhealthy eating habits and consequences, such as obesity. Based on such example, consumer perceptions can be manipulated because they make food-consumption choices based on subjective utility depending on scarcity or abundance situations.

Commercialization of agriculture has been suggested as a viable means for addressing farmer's welfare in urban and peri-urban areas of the developing world. In this regard, Mutsami and Karl (2020) argue that the commercialization of crops and large livestock is an established intervention to solve problems of poverty and food security. Critically, this study focuses on the commercialization of rabbits with a focus on other underlying factors, such as education, health, and micro-credit access, and its implication on tourism poverty alleviation. It finds that rabbit commercialization in urban and peri-urban communities reduces poverty, reduces family size, and enhances education attainment and access to credit. Having looked at studies that establish the relation between food security and poverty, the former enhances individual general well-being, such as health, academic achievement, and financing options for growth.

Global governance mechanisms under the auspices of the United Nations Sustainable Development Goals (UN SDG) focus on the eradication of hunger through food security and safety measures.

According to Vågsholm et al. (2020), the balance between sustainability, food security, and safety as well as prudent utilization of available food is vital in sustainable food production and consumption. Strategies in this regard involve intensive food production, enhanced cycle production, recycling, and reuse of food. In addition, they argue that increased consumption of plant as opposed to animal proteins and reduction of antibiotic use in animal and plant husbandry is key to promoting food safety. They further acknowledge that product labeling is a way forward to increase consumer choice depending on their sensitivity to food wastage.

### Psychological Capital in Food Governance

Social and psychological capital is key to transforming poor migrant communities from penury to self-sustaining individual development. Modesti et al. (2020) carried out a study on the role of social and psychological capital concerning social enterprises with a migratory background (SEMB) in Italy. According to the study, migrant-led social enterprises have a direct contribution to refugee integration in host communities. In this regard, they conclude that social and psychological capital is significant internal capital that facilitate refugee settlement and the ability to turn their adversity into resources for their development. Despite these findings, the role of SEMB in enhancing a migrant community's social integration and effectiveness was left for future research. As such, this study notes gaps in social and psychological capital as a transformative force in food governance concerning security and safety.

In the era of climate change, a policy environment that supports climate-smart options has been promoted as an intervention in reducing global warming. For Lewis and Rudnick, smart agriculture is based on three key concerns, namely (a) contributions to greenhouse gas emissions; (b) susceptibility to anthropogenic effects of climate change; and (c) the nexus between agricultural production, incomes, and food security. Drawing from California state climate-smart policies and programs, that policy must offer sufficient trade-offs between the three concerns for prioritization or reconciliation of the same. California's Climate Smart Agriculture (CSA) allows for mitigation, adaptation, and production agricultural productivity measures depending on resource usage and sustained productivity. Through stakeholder participation, farmers in California have adopted climate action farming practices based on state-sponsored incentive programs.

Social co-governance involving diverse stakeholders is a very crucial component of food security vulnerabilities. Chen and Wu (2019), therefore, define the collaboration between “government, industry, and society” (p. 1) as social co-governance. In this regard, they argue that collaboration between the three must be built on positive psychological capital. Giving the example of pork production in China, they propose four approaches for positive psychological capital–based food safety governance: (a) manufacturers instilling food safety and security values in employees, (b) the government appealing to the sensibilities of food producers and social actors for efficacy toward this regard, (c) the state also publishing food safety standards and guidance for producers, and (d) social persuasion of social actors through participatory approaches in policy formulation.

## Conceptual Framework

Globally, the human population is growing, creating demand for sustainable food safety, security, and health. Accordingly, the UN SDG acknowledges the challenge of hunger and the need to sustainably feed 10 billion people with nutritious and healthy food (Hunter et al., 2016). Presently, food governance systems fall short of enhancing food security at a time when more than 750 million people are hungry and malnourished. According to studies, the global population will hit the 10 billion mark by 2050, generating an urgent need for global and national food security. Against this background, consumer-oriented policies and standards aimed at sustainable consumption are significant.

Notably, the social co-governance model based on stakeholder positive psychological capital is an approach suitable for analyzing food security and tourism poverty alleviation drawing on lessons from China (Wu et al., 2020). In this regard, the four ingredients of the social co-governance model are transplanted into the study of the food governance relationship chain involving national, industry, and enterprise practice seeking to alter consumer behavior and perception toward sustainable consumption for tourism poverty alleviation.

In sum, this conceptual note addresses psychological social co-governance in the developed world, citing examples from China and other developing countries. For this reason, the structure of this study explores industry, enterprise, and national food governance levels separately and as inter-relationships. Specifically, it explains how food governance stakeholders in the developing world may alter consumption patterns toward food security and, subsequently, tourism poverty alleviation. Here below is a diagrammatic representation of the concept applied for the study.

Psychological capital psychological Capital.

### National Food Security Governance

Successful economies build their quest for socioeconomic development policy on food security for its population as well as the safety of the same. China built its economic prosperity on the back of ensuring a food-secure nation with policies derived from experiences from past famine (Browning et al., 2019). Governments play a significant role far as national food governance in terms of sustainable consumption (Beveridge et al., 2019). Consequently, the 1950's famine inspired President Deng Xiaoping's food sufficiency policy in the 1980's, which focused on quantity. Moreover, China has social welfare and pension policies that cushion the poor and vulnerable, hence, sustaining quantity food consumption (Browning et al., 2019). Through this approach, the government takes a welfare approach to the alleviation of the effects of poverty in rural and urban China.

China's socio-cultural orientation as a product of ethical traditional systems influence a consumer clean meat–eating preference; nonetheless, the national policy is centered on safety and economics. Overall, China's government prioritizes agricultural technology, food safety, and security (Garcia et al., 2020). As a result, such policy approaches arouse a Chinese middle-class preference for clean and plant-based meat (Garcia et al., 2020). With food safety incidents on the rise, there is a rising interest in organic food among the aforementioned socioeconomic groups in urban areas. This can be attributed to policy gaps upon which consumer cognition influences market demand for organic food. Similarly, policy lacunas drive unsustainable procurement of endangered sea-cucumber consumption (Fabinyi et al., 2017). As such, both policy action and inaction may sanction consumer cultural orientation even if consumer preference ignores sustainability.

China's practice and policies target small farms with vaccination programs against swine fever, which can decimate small pig farms economically and dent supply of its important pork market. With China being the largest global largest pork producer and consumer, the African swine flu pandemic threatened smallholder farmer incomes and raised consumer safety concerns (Garcia et al., 2020). As a result, a local animal immunization policy targeting small- and medium-scale pig farms sustains an economic sector and consumer safety perceptions to support uptake. The internal market negative psychological concerns on food safety in the largest global pork consumer will, in turn, reduce poverty.

Sustainable interventions modeled on behavioral research guide policy formulation. Behavioral science has been identified as an approach for construing climate change adaptation with regard to sustainable management of scarce environmental resources, such as food, energy, and water (Moore and Boldero, 2017). Eradication of this threat to a critical sector of the Chinese economy calls for development of vaccines with the help of the scientific community and sound government policy. Notably, swine fever is a threat to China's food security being the largest pork producers and consumers, meaning it is a critical part of the country's dietary needs (Garcia et al., 2020). The immunization policy is a safety guarantee that is critical to pork consumption, food security, and small-scale farmers' incomes and, hence, tourism poverty alleviation.

Tourism poverty alleviation strategies through food security policy measures entail the development of standards for land use and small farmers' entrepreneurship perceptions. With regard to food production, a rural land use policy within a local and socioeconomic context relieves the extremes of food pressure and the anthropogenic effects of climate change (Reay et al., 2020). The aforementioned is achievable if the focus is trained on policy-driven lifestyle changes that are based on cognition. Studies have shown that in China's positive spillover effects on environmental sensitivity are fluid compared to Brazil and Denmark (Capstick et al., 2019). A good example is China's agricultural entrepreneurship policy, which transforms cognition of the value of agriculture as a business. As a result, about 4.5 million farmers moved back to start their businesses to diversify income sources (Kong et al., 2019). Being an established agricultural economy with near sufficient food resources, an entrepreneurship approach is suitable for stabilizing incomes and reducing poverty.

Overall, evidence-based policy initiatives based on education and cultural interventions are critical for tourism poverty alleviation and sustainable consumption. Although China's consumption practices are largely market-driven, Western perceptions of reducing health and environmental consequences of consumption are education and prevention programs (Carrus et al., 2018). Certainly, China can draw lessons from the West for evidence-based policy options for its intervention against environmental and health problems of consumption. Some consumption and tourism poverty alleviation efforts can be induced through policies that induct learners on conserving cues to influence behavior (Watson et al., 2017). Consequently, the policy has the potential to influence consumption through the evidence-driven interventions for healthy consumption, environmental safety, and food security, which, in turn, alleviates poverty.

Policy inaction directly influences market-driven consumption based on developing individualist cultures in developing China with an impact on the market value chain. A rising urban population's cumulative demand preferences create a chain of producers, middlemen, transportation, and other sectors whose productivity is a critical poverty cushion. Social norms and cultural practices are directly linked to food choices, which then impact well-being and health outcomes (Carrus et al., 2018). In addition, long-held cultural dietary needs also contribute to a generic effect in organic food consumption, which sustains the economic sector. Despite the benefits of a non-regulated food security environment, widely held practices may only expand sustainably through policy support.

### Industry Initiatives

National policy standards with a safety focus may also be part of corporate social responsibility (CSR) or industry safety and health standards practice to influence consumer choice. For the Chinese pork industry, the China Brand Name Association certifies products as voted trust brand (VTB) to assure consumers of their safety, reliability, quality, and affordability (Wu et al., 2020). Generally, this is dependent on internal and external factors that influence consumer decisions on food purchase behavior (Martínez-Ruiz and Gómez-Cant, 2016). Factors that producers may use to attract consumer attention include product farming practices, production methods, and nutrition (Wu et al., 2020). When these values are instilled in employees in the production process, they affect consistency and consumer behavior as far as choice is concerned.

Industry labeling practices for food safety and security promote consumer confidence in products if sustainable consumption key messages are embedded in. Shifts in socio-cultural practices toward individualism increase consumer psychological orientation toward healthy and sustainable choices (Carrus et al., 2018). In China, the organic food industry in big cities is a response to the increasing concern about the use of pesticides and antibiotics (Liu and Zheng, 2019). Consequently, the industry categorizes organic and inorganic food, traditional, and pollution-free, among others, to win consumer trust in the food's safety and nutritional value (Liu and Zheng, 2019). The market-generated responses to food safety and sustainable consumption through classification alters consumption patterns in response to their safety concerns.

Organic farming initiatives in the face of increasing demand for dairy products in China increase crop yields to meet emerging consumer choices and incomes for dairy farmers. As evidence shows, dairy farming is an alternative organic solution to increasing crop yield and replaces synthetic and environmentally unsustainable synthetic fertilizers (Fang et al., 2020). Organic food classification labeling not only distinguishes organically produced products to influence the growing consumer preference, but also provides additional income to dairy farms to play a role in organic food production (Liu and Zheng, 2019). Here, consumer trust and attitudes toward organic foods is a key support system for small and medium-scale dairy farmer recycling efforts (Lazaroiu et al., 2019). Importantly, the industry in China is responding to new and modern consumer interest in sustainable organically produced foods, which creates a value chain for organic fertilizer for the benefit of small-scale dairy producers.

Industry plays a critical role in encouraging organic food consumption through consumer cognition and urban affordance. Research has demonstrated that individuals respond to cues that sometimes lead to obesity or healthy weight consciousness (Watson et al., 2017). Pavlovian cues in response to advertising is a learning mechanism that differs between obese and healthy-weight individuals (Watson et al., 2017). The industry may capitalize on the effects of external influences on consumer decisions on food attributes and consumption. China's nascent organic industry may capitalize on its specific social context with regard to healthy production and consumption and the value chain it supports (Martínez-Ruiz and Gómez-Cant, 2016). All this may be captured on industry standards of advertising to divert people to organic food production and consumption for food security and tourism poverty alleviation among organic farmers.

Industry practices geared toward sustainable food production also draw from government regulatory initiatives on sustainable consumption. Japan's conservative government policy on whaling supports industry efforts toward sustaining its internal whale meat market (Butler-Stroud, 2016). Forces driving its consumption derive from the national government, diet, and the general society. The government subsidizes the industry to meet the demands of traditional whale meat consumers and commercialize the industry (Butler-Stroud, 2016). The fact that it has withdrawn from multilateral governance structures influences national consumption to create jobs in the whaling industry.

Fundamentally, Communist government policy support and industrial practice toward food security have the potential to alter poverty and sustainable consumption. For this to take effect, it requires significant staff and organizational change, which then cascades to collective industry practice toward sustainable practices (Lee et al., 2017). Practical lessons in this regard are pro-environmental value orientations that determine collective action (Lee et al., 2017). The same standards may be applied with regard to changing attitudes in urban areas toward organic foods in urban China. As such, an increased consumption of an organic food industry standard may extend their practices in the organic food production sectors. Most definitely, the concentration of production to meet a rising and expanding urban behavior–driven demand has the potential to increase employment and, thus, poverty reduction.

### Enterprise Initiatives

The role of enterprise may combine with industry standards through collaborative approaches to climate-sensitive use of marine food resource management, such as fish stocks. Climate change effects such as rising sea levels and other anthropogenic effects threaten fish stocks and, hence, cause food security of small-scale farmers (Butler et al., 2020). In Indonesia and Papua New Guinea, small-scale farmers' contribution to food security in the isolated Asia Pacific Islands depend on integrated systems that rely on the distributor and consumer feedback that inform policy (Butler et al., 2020). In particular, co-governance mechanisms at the village level bring together consumers and fisheries in a bottom-up approach to policy making and consumption practices (Butler et al., 2020). Participatory approaches, inculcate ownership, and responsible consumer choices that are directed toward sustainable consumption safeguard the future food security generations.

Entrepreneurs play a pivotal role in influencing dynamics of consumer preferences and the procurement chain from producers. Supermarkets in Vietnam have increased variety and choice and spending habits on food items depending on incomes (Trinh et al., 2020). Accordingly, regions with high consumption of fat and processed carbohydrates experience an impact quality with negative impacts on health. In this regard, noted evidence of household consumption of high-fat and high-protein foods contribute to healthy eating habits among consumers. Similarly, India's policy bars biofortification of rice, which affects any dietary consumption among predominantly poor consumers. Once again, government policy constrains enterprise efforts to wean societies of dependence on native rice (Bashir et al., 2013). Under such circumstances, the pursuit of health outcomes among rice farmers through fortification constrains the role of enterprises.

Bold and inclusive measures are capable of making bold collective interventions for sustainable food consumption and tourism poverty alleviation. In this context, one finds the complex interplay of interests between food security and mitigating the effects of climate change (Ziervogel and Ericksen, 2010). India's approach of cash transfer safety nets to vulnerable households mitigates against the food security effects of climate change. Unfortunately, the cost of maintaining household asset-building programs are exorbitant and unsustainable (Ziervogel and Ericksen, 2010). Consequently, there is a combination of formal and informal components of food security and tourism poverty alleviation. An appropriate informal solution for a developing economy capitalizes on social rather than technical innovations to generate a self-sustaining micro-economy. On the other hand, the formal sector may address the capital needs against the backdrop of policy supported micro-loans to enhance food production and consumption among the poor (Ziervogel and Ericksen, 2010). This is tantamount to investment in already existing local economies through state and enterprise efforts.

Enterprises founded on goal-oriented frameworks are critical in creating environmentally sustainable food consumption for tourism poverty alleviation. They do this through the promotion of background knowledge and provision of information on sustainable consumption cues at the point of sale, which have been identified as possible interventions (Vermeir et al., 2020). The challenge with consumption cues is that they are usually ignored at the point of purchase although sometimes climate-sensitive food products may be costly. Industry, government, and enterprise may collaborate to alter consumer perception toward sustainable consumption (Vermeir et al., 2020). Perhaps developing economies need to complement this effort with the policies that make sustainable production cost-effective and key messaging to stimulate green consumption behavior. This will go a long way in promoting goal-oriented behavior through collective policy, industry, and enterprise to influence the same and increase incomes among organic producers and the value chain it supports.

## Conclusion

This paper interestingly uses the concept of food governance as poor people's psychological “healing” mechanism when shopping for food with a purpose of safety. Conceptual analyses from different levels and aspects of people's livelihood governance and poverty remedies offer logical and evidenciary supports for the authors' arguments and call for more governmental efforts. The good thought is that food safety governance can bring psychological safety and, thus, generate a feeling of wellnesse. This is a piece that offers hints for governmental units to go “in deep” to the governed people's minds in a context of livelihood consumption—a piece that utilizes micro-psychological dynamics to generate implications for macro-governmental policies.

To sum up, food governance mechanisms in developing nations have a direct implication on consumer preferences. So far, the concept reveals that there are competing needs that fall short of the trade-offs required to balance food safety, security, and sustainable consumption thereof. National policies establish standards and procedures that alter consumption for tourism poverty alleviation. Similarly, coordinated efforts between industry and enterprise facilitate linkages with national systems in implementation and regulatory standards geared toward consumer preferences. For instance, China's clean meat and pork consumption habits are a reflection of its evolution of middle-class consumer choices with a focus on organic consumption. In this regard, immunization and recycling are critical components in food governance measures to safeguard the small scale farmers' economic interests.

Developing countries are hardly aligned to capitalize fully on positive psychological capital. Most developing countries exist in evolving food markets with divergent consumer preference toward organic foods, bio-fortification, and sustainable marine resource exploitation. Primary indications show that total consumer total preference creates a need for research on consumer safety and policy for tourism poverty alleviation. Despite these concerns, positive psychological capital can facilitate interaction between national systems, industry, and enterprise. Consumer-driven systems, such as in China, addressing food security and safety are the fulcrum around policies and market practices that safeguard the majority of small farmers who are critical in enhancing food governance. As the production chain emphasizes safety and health factors, consumers trust. Future studies can expand the discussions in this article to a variety of sub-contexts under tourism poverty alleviation, such as tourism. For example, tourists' psychological capital facilitated by food safety through well-designed governance may lead to more consumption to stimulate economics and partially contribute to tourism poverty alleviation.

## Author Contributions

GH collected the data set, wrote the original paper, and analyzed for the results. KL-L conceptualized the main theme, reviewed and edited the manuscripts, and is responsible for the interactive review process.

## Conflict of Interest

The authors declare that the research was conducted in the absence of any commercial or financial relationships that could be construed as a potential conflict of interest.
